# Adsorption of SF_6_ Decomposed Products on ZnO-Modified C_3_N: A Theoretical Study

**DOI:** 10.1186/s11671-020-03412-y

**Published:** 2020-09-25

**Authors:** Peng Wu, Xiaoxing Zhang, Dachang Chen, Ju Tang

**Affiliations:** 1 School of Electrical Engineering and Automation, Wuhan University, Wuhan, 430072 China; 2 Hubei Key Laboratory for High-efficiency Utilization of Solar Energy and Operation Control of Energy Storage System, Hubei University of Technology, Wuhan, 430068 China; 3 State Key Laboratory of Power Transmission Equipment & System Security and New Technology, Chongqing University, Chongqing, 400044 China

**Keywords:** C_3_N monolayer, Metal oxide modification, Density functional theory, SF_6_ decomposed species

## Abstract

SF_6_, as an outstanding insulation medium, is widely used in the high-voltage insulation devices, guaranteeing the safe operation of the power system. Nevertheless, the inevitable partial discharge in a long-running device causes the decomposition of SF_6_ and deteriorates its insulation performance. In this work, DFT calculations were performed to study the adsorbing and sensing properties of ZnO-modified C_3_N (ZnO-C_3_N) nanosheet towards SF_6_ decomposed products, in order to propose a novel nano-candidate for evaluating the operation status of SF_6_ insulation devises. We first investigated the structure of ZnO-C_3_N monolayer and then simulated its adsorption behaviour upon four typical SF_6_ decomposed species, namely H_2_S, SO_2_, SOF_2_, and SO_2_F_2_. It is found that the ZnO-C_3_N monolayer can exhibit desirable reactivity and sensitivity on SO_2_, SOF_2_, and SO_2_F_2_, leading to the intense deformation of gas molecules and large adsorption energies. These consequences allow the potential application of gas adsorbent based on ZnO-C_3_N monolayer for removing impurity gases from SF_6_ insulation equipment. According to the analysis, it is supposed that ZnO-C_3_N monolayer is qualified to be used in maintaining insulation strength and ensuring the safe operation of power system.

## Introduction

With the rapid development of nanotechnology, the application of sensors based on novel nanomaterials is increasing in recent years. By virtue of its quick response, low consumption, low cost, and high sensitivity, nano-sensor has been exclusively studied in the field of medical, military, gas detection and environment monitoring [1–4]. Resistance-type sensor, as one of the most commonly used sensors, is favoured by scholars because of its simple structure and working mechanism. In the early stage, the graphene is an attractive material in gas detection for its excellent performance on physical and chemical, such as large specific surface area, high carrier mobility, and favourable heat conductivity [5–8]. However, the graphene is limited in the application of gas recognition due to its zero bandgap characteristic [9, 10], underperforming in identifying common gases like CO, CO_2_, CH_4_, N_2_, NO_2_, NH_3_, H_2_, and H_2_O [11]. Afterwards, with the joint effort of scholars, numerous novel nanomaterials with the same properties to graphene but free from zero bandgap have sprung up in the field of gas sensing, including but not limited to transition metal dichalcogenides (TMDs) [12–14], metal carbides and nitrides [15], layered group III-VI semiconductors [16, 17], and group III-V nitrides [18–20].

Among the new emerged graphene-like materials, C_3_N is synthesized by the direct pyrolysis of hexaaminobenzene trihydrochloride single crystals or the polymerization of 2, 3-diaminophenazine [21, 22], which has attracted considerable attention as a gas adsorbent [23–25]. The C_3_N is intrinsically an indirect semiconductor with the bandgap of 0.39 eV that can be tuned by fabrication of quantum dots with different diameters [22]. In micro appearance, C_3_N can be regarded as a 2 × 2 graphene supercell substituted by two nitrogen atoms, possessing a planar honeycomb lattice with six carbon atoms and two nitrogen atoms. As a result of the added N atoms, the intrinsic C_3_N shows stronger chemical activity and higher carrier mobility but keeps similar structural stability compared to graphene, making the C_3_N monolayer a competitive candidate for gas detection [26]. In terms of the adsorption ability, researchers have proved that the intrinsic C_3_N has excellent selectivity and sensitivity [27] in detecting NO_2_ and SO_2_, while for other gases, there is no obvious adsorption effect. Nevertheless, research makes clear that the surface reactivity of C_3_N could be largely promoted by the modification of impurity particles. For instance, Pashangpour and Peyghan [28] carried out a comparative experiment on CO adsorption ability of intrinsic and doped C_3_N nanosheet; their results illustrate that Al dopant can bring about much stronger binding interaction than the pristine C_3_N. Later, Zargham Bagheri [29] theoretically studied Si-doped C_3_N for adsorption of acetone, and it is found by replacing a C atom with a Si atom, adsorption energy can increase from − 9.7 to − 67.4 kcal/mol, and the sensitivity increases as more C atoms are substituted.

Metal oxide is a commonly used alternative in surface-modification to enhance the chemical reactivity for gas interactions. As one of the metal oxide semiconductors, ZnO has a bandgap of about 3.37 eV with exciting binding energy of about 60 meV, chemical stability, excellent photocatalytic properties, and high activity to some specified gases [30]. According to Ref [31], ZnO can grow in 0-dimensional (0D), 1-dimensional (1D), and 2-dimensional (2D) nanostructure morphologies, with examples of each class including nanoclusters, nanowires/nanotubes, and nanosheets/nanoribbons, respectively. Given the characteristic of easily controlled size and morphology [32, 33], ZnO nanoparticle is a promising material in working as a dopant to improve the sensing performance of nano-surfaces [34–36]. Recently, a few scholars have proposed theoretical studies on improving surface activity of nanomaterials by using single-molecule metal oxide dopant. E. Mohammadi-Manesh et al. [37] investigated the adsorption ability of Cu- and CuO-decorated graphene upon H_2_S theoretically and found the conductivity of the modified graphene changed significantly compared to that of intrinsic graphene after the adsorption of H_2_S. Asadi and Vaezzadeh [38] designed a B- and CuO-decorated graphene sheet for detecting H_2_S and CO based on density functional theory (DFT). The simulation in these works was carried out by DFT and its computational codes extend the atomic or molecular structure periodically based on the defined supercell and then calculate the physical properties of the entire system. Based on this method, the adsorption of an atom or molecule on the substrate as a sensor is used to study nanostructures. The foregoing reports stimulate us to perform related and further research on this topic about single metal oxide molecule doping; herein, we doped C_3_N with ZnO molecule instead of ZnO nanoparticle as a simplification to explore the effect of ZnO on gas sensing.

SF_6_ is a widely used medium in gas insulated switchgear (GIS) with prominent insulating and arc-extinguishing properties [39]. The inevitable accident inner defects, such as partial discharge (PD) in GIS, will decompose SF_6_ to some low-fluoride sulphides such as SF_4_, SF_3_, and SF_2_ [40]. These by-products would further react with the trace moisture and oxygen, generating some stable chemicals such as H_2_S, SO_2_, SOF_2_, and SO_2_F_2_ [41]. The insulating reliability of these by-products is much lower than SF_6_ and their existence will accelerate PD evolution if left alone. Therefore, so as to guarantee the safe operation of GIS, it is essential to detect or sweep away these gases. In this paper, we chose ZnO as a dopant and built the model of ZnO-modified C_3_N (ZnO-C_3_N) monolayer to study its adsorption performance upon typical SF_6_ decomposed species (H_2_S, SO_2_, SOF_2_, and SO_2_F_2_) theoretically. By analysing the structural changes, electron transfer behaviour, band structure, and density of state (DOS), the impact of ZnO dopant on interaction between the C_3_N surface and gas molecules were comprehensively studied. The purpose of our work is to give detailed adsorption and sensing mechanism of ZnO-C_3_N monolayer for potential application to detect or scavenge the impurity gases in the SF_6_ insulation devices.

## Computational Details

All of the calculations based on DFT were carried out in Dmol^3^ package [42]. For the sake of better describing the non-uniform electron density of realistic system, we employed the generalized gradient approximation (GGA [43]) within the Perdew-Burke-Ernzerhof (PBE) function and the dispersion correction of TS to deal with the electron exchange-correlation terms [44, 45]. The DFT semi-core pseudopots (DSSP) was induced for core treatment and double numeric basis with polarization (DNP) was chosen as the atomic orbital basis set [46]. Monkhorst-Pack *k*-points of 6 × 6 × 1 meshes were defined in both geometric optimization and electronic structure calculations [47]. The energy convergence tolerance, maximum force, and maximum displacement in geometric optimization were respectively set as 1.0 × 10^−5^ Ha, 0.002 Ha/Å, and 0.005 Å [48]. Besides, the monolayer and its neighbouring image were separated by a vacuum spacing of 15 Å to avoid the interaction between them.

**Fig. 1:**
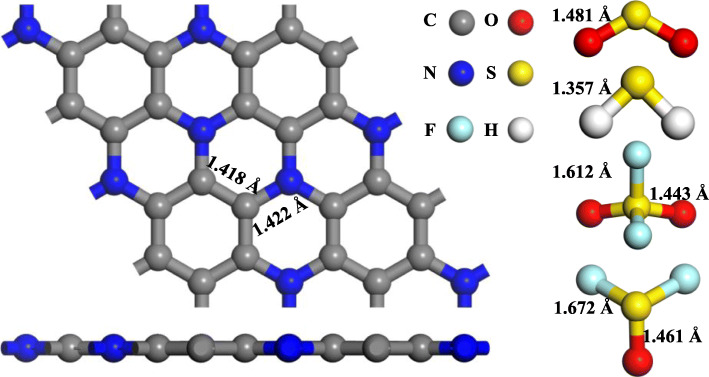
Optimized structure of C_3_N monolayer, H_2_S, SO_2_, SOF_2_, and SO_2_F_2_

As illustrated in Fig. 1, the 2 × 2 × 1 supercell of C_3_N monolayer and the gas molecules were established and optimized prior to the doping and adsorption process. The C-N bond (1.422 Å) in the optimized monolayer is slightly longer than C-C bond (1.418 Å) as a consequence of the larger radius of N atom in comparison with C atom. The lattice constant obtained in this work is 4.92 Å, similar to the reported results in Ref. [25, 49]. We calculated the charge transfer between the molecule and monolayer by Hirshfeld analysis and defined *Q*_T_ to represent the charge change in the gas molecule. A positive *Q*_T_ indicates the electron-releasing behaviour of the gas molecule. Oppositely, it suggests the electron receiving behaviour of the gas molecule.

## Results and Discussion

### Analysis of ZnO-Modified C_3_N Monolayer

**Fig. 2:**
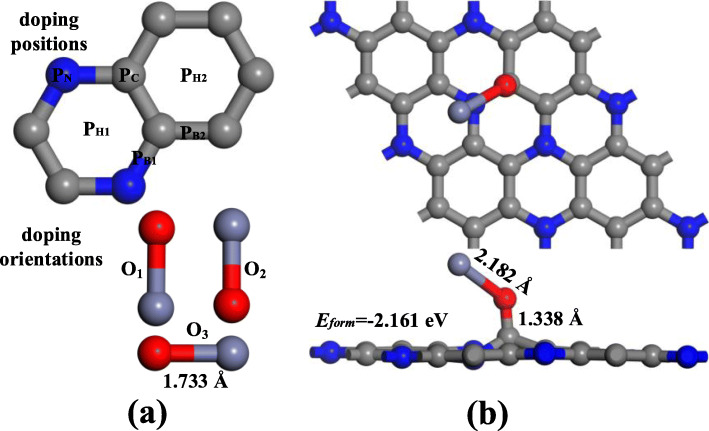
**a** The approaching positions and orientations of ZnO molecule. **b** The most stable configuration of ZnO-C_3_N

After geometric optimization, the ZnO was placed on the surface of C_3_N monolayer in different orientations and position to explore the most reasonable configuration of ZnO-C_3_N. According to Fig. 2a, ZnO particle is approaching C_3_N monolayer through the vertical (O_1_, O_2_) and parallel (O_3_) orientations to the plane at the position of the centre of the hexagonal structure (P_H1_, P_H2_), the middle point of the C-C and C-N bonds (P_B1_, P_B2_), and right above the C atom (P_C_) and N atom. We defined formation energy (*E*_form_) to assess the stability of ZnO-C_3_N monolayer, calculated as follows:

1 $$ {E}_{\mathrm{form}}={E}_{\mathrm{ZnO}\hbox{-} {\mathrm{C}}_3\mathrm{N}}-{E}_{\mathrm{ZnO}}-{E}_{{\mathrm{C}}_3\mathrm{N}} $$

where *E*_ZnO_ and ${E}_{{\mathrm{C}}_3\mathrm{N}}$ are the energy of ZnO molecule and C_3_N monolayer before doping, and ${E}_{\mathrm{ZnO}-{\mathrm{C}}_3\mathrm{N}}$ is the energy of ZnO-C_3_N structure. When the close-range “bonding” between atoms occurs in the extended atomic structure, the total energy is reduced and resulting in a negative *E*_*form*_ [50]; the structure with the largest *E*_*form*_ is selected for adsorption and further analysis.

**Fig. 3:**
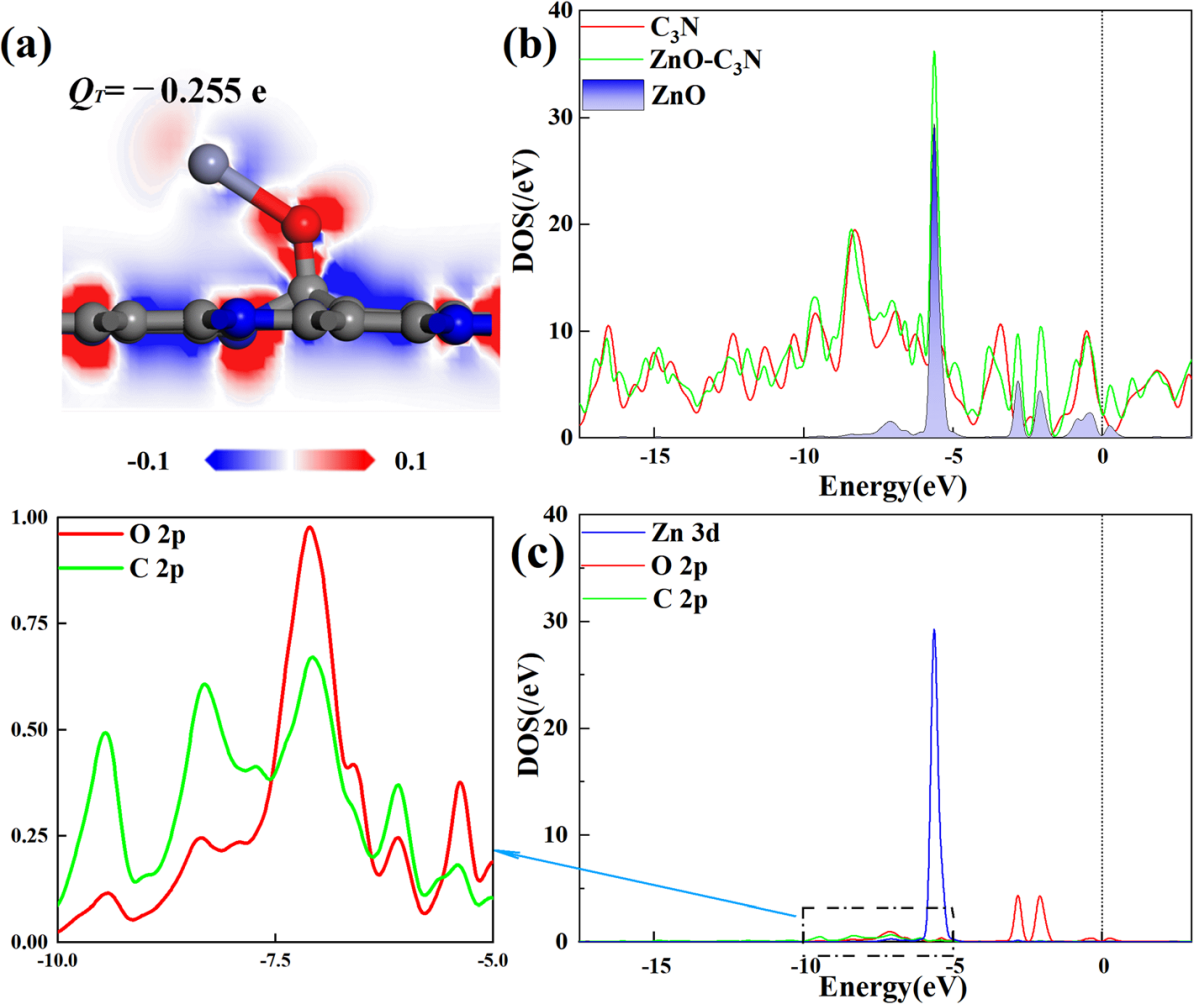
**a** DCD and **b**, **c** DOS and PDOS of ZnO-C_3_N monolayer

All the configurations of ZnO-C_3_N monolayer are displayed in Figure S1, S2, S3. These results demonstrate that most of the structures with large *E*_*form*_ are in O_2_ orientation and the ZnO particle prefers to approach the C_3_N surface by O-oriented position and trapped by a C atom. Furthermore, each modification process in this study is spontaneous since the *E*_*form*_ is negative and the maximum *E*_*form*_ is obtained by placing the ZnO particle at S_C_ in O_2_ orientation. As can be seen in Fig. 2b, the diatomic molecule is attached to C_3_N with a tilt of 40°. The Zn-O bond is elongated from 1.733 Å to 2.182 Å and the C-O bond is measured as 1.338 Å. Under the effect of ZnO, the surface of C_3_N is no longer flat but a certain degree of distortion occurs, and meanwhile the C atom nearest to O atom is pulled out of the surface. For further discussion of the electronic behaviour of ZnO-C_3_N monolayer, the deformation charge density (DCD) and density of state (DOS) are depicted in Fig. 3. In Fig. 3a, the red region corresponds to an increase in charge density and the decrease is represented in blue. When ZnO molecule is adsorbed, it extracts 0.255 e from the C_3_N monolayer and a distinct red area can be recognized around the O atom. While the Zn atom is surrounded by a blue area, signifying the difference in electronegativity between O atom and Zn atom. Moreover, the significant raise of charge density between O atom and C atom suggest the formation of C-O bond which can also be supported by the intense hybridization between the states of O 2p orbital and that of C 2p orbital, as shown in Fig. 3c. From the DOS curve in Fig. 3b, it is obvious that the introduction of ZnO leads to an increase in the systemic DOS and the appearance of several novel peaks. It can be identified that the new emerged small peaks are contributed by the O atom at about − 2.5 eV and the biggest one located at − 5.6 eV apparently resulted from the Zn 3d orbital. The changes in DOS and the hybridization between orbitals confirmed the fact that the ZnO particle have firmly adsorbed on the surface of C_3_N and exerted great impact on the electronic structure of the whole system.

### Adsorption Behaviour of ZnO-C_3_N Monolayer

**Fig. 4:**
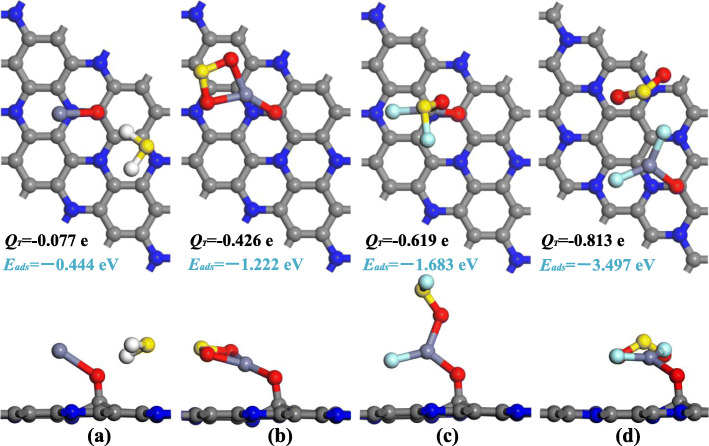
The adsorption configuration of **a** H_2_S system, **b** SO_2_ system, **c** SOF_2_ system, and **d** SO_2_F_2_ system

**Fig. 5:**
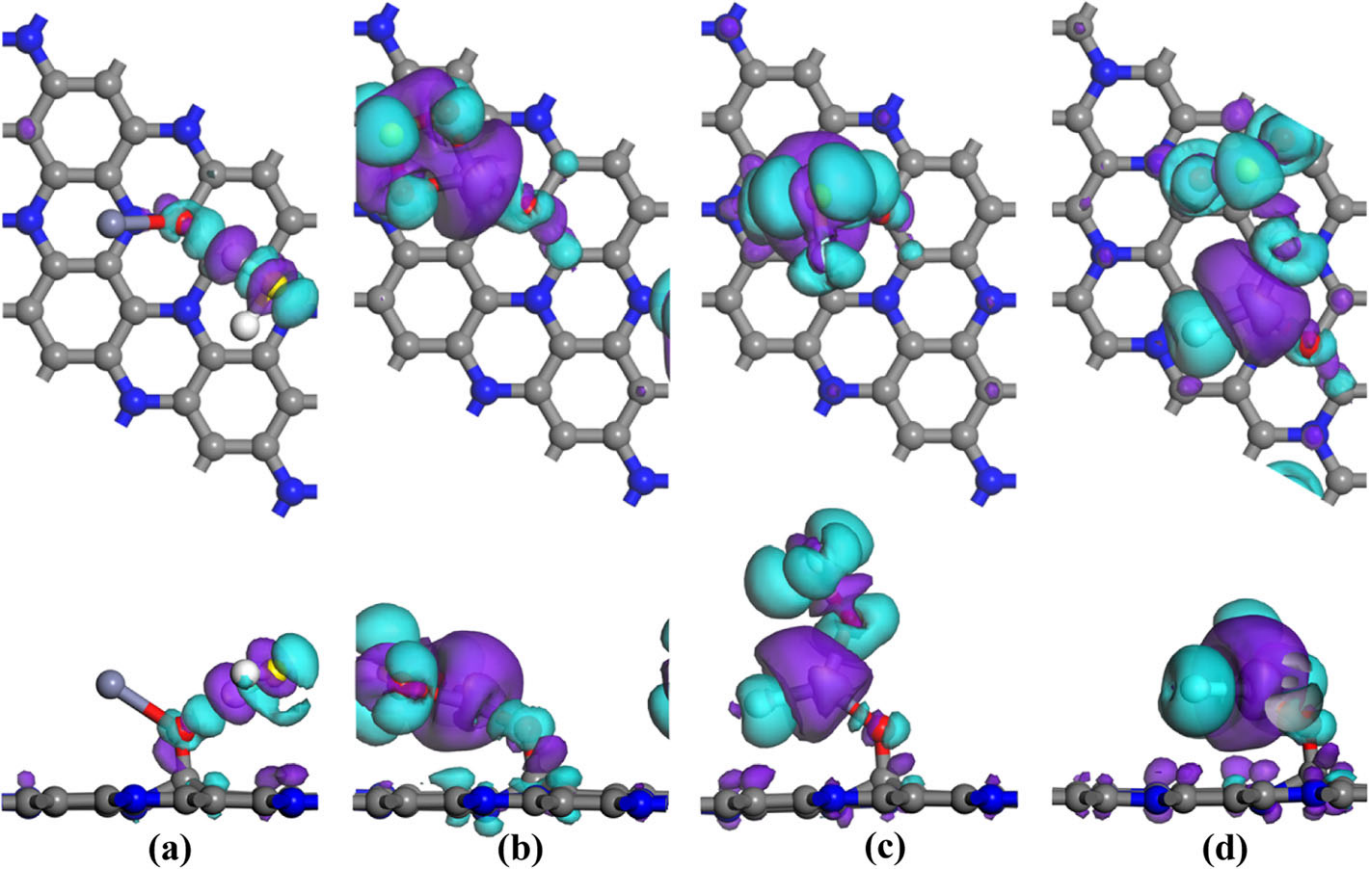
The EDD of **a** H_2_S system, **b** SO_2_ system, **c** SOF_2_ system, and **d** SO_2_F_2_ system

To fully compare the possible adsorption parameters and select the most desirable configuration for analysis, we put each gas molecule above the surface of ZnO-C_3_N monolayer in various orientations. For example, for triatomic molecules, namely H_2_S and SO_2_, we made the plane composed of the three atoms parallel or vertical to the surface with the S atom upward or downward. The adsorption energy (*E*_*ads*_) is employed to describe the energy changes of different adsorption structures and calculated as

2 $$ {E}_{\mathrm{ads}}={E}_{\mathrm{ZnO}\hbox{-} {\mathrm{C}}_3\mathrm{N}/\mathrm{gas}}-{E}_{\mathrm{ZnO}\hbox{-} {\mathrm{C}}_3\mathrm{N}}-{E}_{\mathrm{gas}} $$

where *E*_gas_ and ${E}_{\mathrm{ZnO}-{\mathrm{C}}_3\mathrm{N}}$ are the energy of the isolated gas molecule and the ZnO-C_3_N monolayer before adsorption, ${E}_{\mathrm{ZnO}-{\mathrm{C}}_3\mathrm{N}/\mathrm{gas}}$ represents the energy of the system with gas adsorbed. After the local minimum total energy of each situation was obtained, only the structure with the maximum *E*_ads_ was chosen for further discussion, as given in Fig. 4, and the electron density difference (EDD) is portrayed in Fig. 5 for better understanding of the charge transfer mechanism.

The H_2_S adsorption system is given in Fig. 4a, wherein H_2_S molecule is adsorbed in parallel position and the nearest atomic distance between H_2_ molecule (H atom) and ZnO dopant (O atom) is measured as 2.042 Å. The H-S bond of the capture H atom is elongated to 1.374 Å compared with the 1.357 Å in its isolated state, while the other H-S remains unchanged during the adsorption process. The slight deformation of the geometry configuration suggests the interaction between H_2_S molecule and ZnO-C_3_N monolayer is weak. Combined with the *E*_ads_ (− 0.444 eV) and *Q*_T_ (− 0.077 e), it is clear that H_2_S molecule cannot stably adhere to ZnO-C_3_N monolayer; we assume that ZnO-C_3_N monolayer is unsuitable for detecting H_2_S. For the SO_2_ molecule shown in Fig. 4b, both O atoms are trapped by Zn atom with the distance of 2.020 and 2.031 Å, respectively. The ZnO particle bends closer to the surface and the angle formed by Zn-O-C is reduced from 129 to 118° due to the presence of SO_2_. At the same time, from the Hirshfeld analysis, we find that the S atom acts as an electron donator with a loss of 0.164 e and its adsorption effect to O atoms is weakened, resulting in the extension of S-O bonds from 1.481 to 1.619 Å [51]. Inversely, the strengthened inter-atomic force, because of the electron increase (0.292 e) in the O atom of ZnO, has shortened the Zn-O bond from 2.182 to 1.869 Å. Apart from the geometric variations and electron transfer, the *E*_ads_ up to − 1.222 eV is another evidence of strong interaction during the adsorption process, indicating potential application of ZnO-C_3_N monolayer in detecting SO_2_. As for the SOF_2_ and SO_2_F_2_ adsorption system given in Fig. 4c and d, it can be seen that the target molecules have undergone dramatic change in morphology. The F atom in SOF_2_ gets rid of the constraint of S-F bond and adsorbed by Zn atom at a distance of 1.830 Å. An even more drastic change can be observed in SO_2_F_2_ where both of the S-F bonds broke and formed Zn-F bonds with the lengths of 1.802 and 1.883 Å. In addition to the formation of Zn-F bond, the remaining SOF group in SOF_2_ system is also trapped by the Zn atom through Zn-O bond. But in the case of SO_2_F_2_ system, it is interesting to note that the SO_2_ group generated by the decomposition of SO_2_F_2_ is not captured but keeps a distance from Zn atom, and with its S-O bonds calculated the same as isolated SO_2_ in length. The pronounced deformation of gas molecules is associated with the large *E*_ads_ calculated as large as − 1.683 eV in SOF_2_ system and − 3.497 eV in SO_2_F_2_ system. Based on this, the adsorption of SOF_2_ and SO_2_F_2_ onto ZnO-C_3_N can be determined as strong chemisorption accompanied by a large amount of charge transfer in this process, indicating the possible existence of significant electron orbital hybridization. With the large *E*_ads_ and *Q*_T_, ZnO-C_3_N monolayer can provide more stable adsorption to SO_2_, SOF_2_, and SO_2_F_2_ than other nanomaterials, as listed in Table 1, the adsorption configuration of ZnO-C_3_N monolayer is larger than the listed nanomaterials by 0.358–3.281 eV and 0.038–0.811 e, ensuring the adsorption performance of this material when used in gas detection, whereas we speculate that it is hard for these gas molecules (SO_2_, SOF_2_ and SO_2_F_2_) to get rid of the strong interaction force as a consequence of the large E_ads_. Hence, in order to prevent the performance degradation caused by sensor poisoning, measures such as high-temperature annealing or ultraviolet radiation should be taken to improve the desorption performance of the ZnO-C_3_N monolayer. The specific desorption performance analysis will be provided in the “Gas sensing performance evaluation” section.

**Table 1:** The adsorption configuration comparison of ZnO-C_3_N monolayer and other nanomaterials

Substrate	Gas	*E*_ads_/eV	*Q*_T_/e	Substrate	Gas	*E*_ads_*/eV*	*Q*_T_*/e*
ZnO-C_3_N	SO_2_	**− 1.222**	**− 0.426**	Ni-BNNT [52]	SO_2_	− 0.864	0.105
	SOF_2_	**− 1.683**	**− 0.619**		SOF_2_	− 0.522	0.078
	SO_2_F_2_	**− 3.497**	**− 0.813**		SO_2_F_2_	− 0.223	− 0.035
C_3_N [27]	H_2_S	− 0.230	− 0.004	Ni-ZnO [53]	SO_2_	− 0.245	− 0.086
	SO_2_	− 0.620	− 0.23		SOF_2_	− 0.207	0.016
	NO_2_	− 0.790	− 0.388		SO_2_F_2_	− 0.219	0.003
Au-TiO_2_ [54]	SO_2_	− 0.657	− 0.156	Au-MoS_2_ [55]	SO_2_	− 0.946	− 0.222
	SOF_2_	− 0.593	− 0.006		SOF_2_	− 0.332	− 0.095
	SO_2_F_2_	− 0.200	0.039		SO_2_F_2_	− 0.175	0.002

In terms of the EDD shown in Fig. 5, the blue part indicates the electron accumulation region and the other part in purple is the electron depletion region. For H_2_S system, a small accumulation region can be found between H atom and O atom, while most of the accumulation and depletion regions are located around the H_2_S molecule, suggesting the small charge transfer and the redistribution of molecular orbitals in H_2_S molecule. In the SO_2_ adsorption system, there are obvious depletion regions that surround the S atom and Zn atom, whereas the accumulation regions are mainly distributed around O atoms and above S atom. This phenomenon confirms the electron receiver role of SO_2_ molecule, in accordance with the *Q*_T_ (− 0.426 e) obtained from Hirshfeld analysis. In SOF_2_ and SO_2_F_2_ systems, judging from the large scale of accumulation and depletion regions, there are remarkable charge transfers and electron hybridization in both systems. The accumulation regions are distributed among the atoms of the target gas, while the depletion regions are mainly localized around Zn atom, reflecting the distinct electron-donating property of Zn atom. As a result, these electronic behaviours make the assumption that the ZnO-C_3_N monolayer has strong adsorption to the gas molecules more persuasive.

### Electronic Properties of ZnO-C_3_N Monolayer on Gas Adsorption

**Fig. 6:**
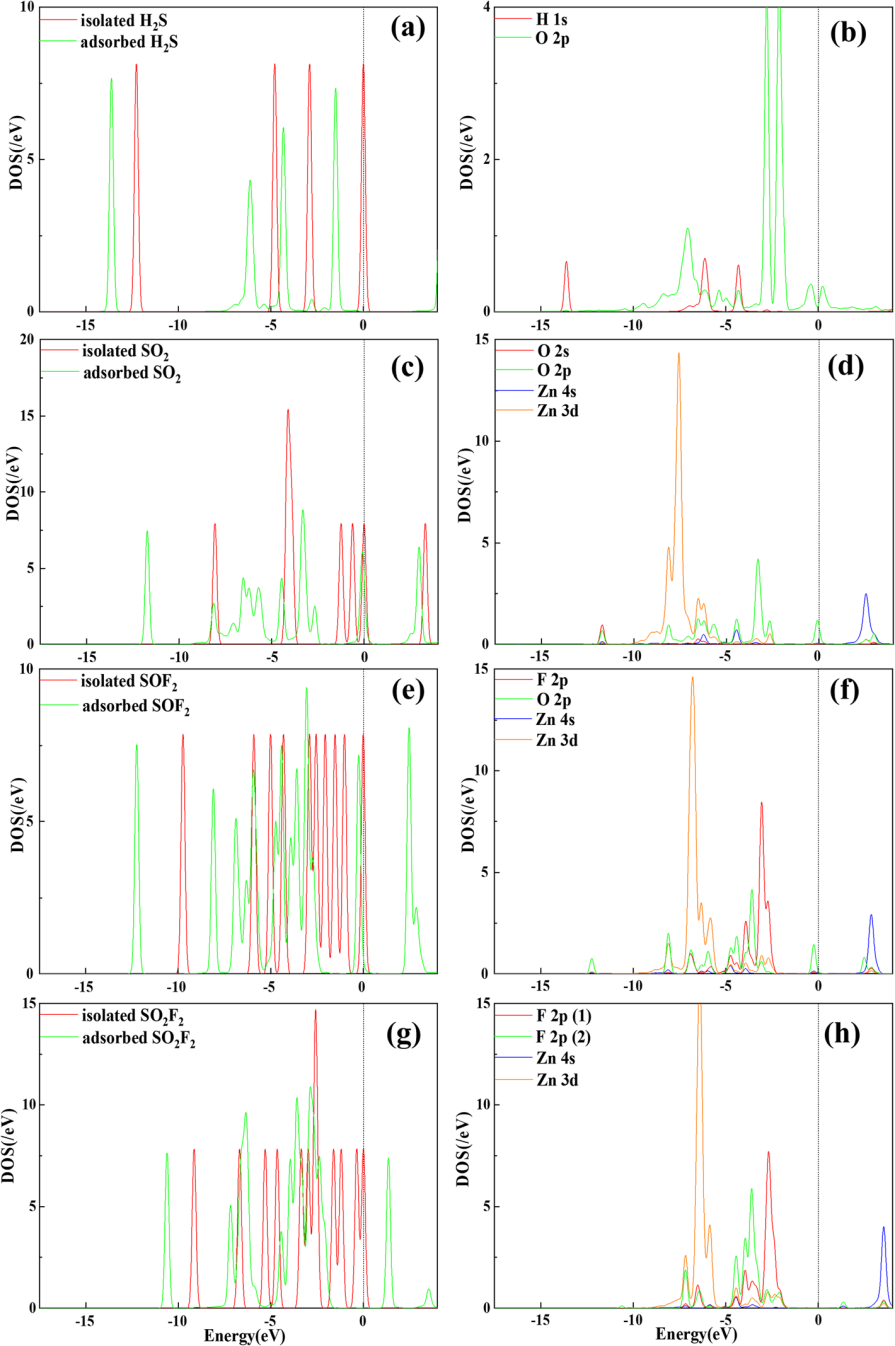
DOS and PDOS of the gas molecule adsorbed on ZnO-C_3_N monolayer. **a**, **b** H_2_S system. **c**, **d** SO_2_ system. **e**, **f** SOF_2_ system. **g**, **h** SO_2_F_2_ system

As reported in graphene- [56], SWCNT- [57], and MoSe_2_- [58] based gas sensing researches, DOS is another important parameter in investigating the electronic behaviour between gases and nanostructure. It can be seen in Fig. 6a that the redistribution of the molecular orbitals in H_2_S is in accord with the conclusion derived from the EDD in Fig. 5a. The hybridization between the H 1s and O 2p orbitals is available near − 4 and − 6 eV but of a low degree, demonstrating the weak interaction and the tiny possibility in forming a new H-O bond. As to the SO_2_ system in Fig. 6c, the antibonding orbital slightly moves near the Fermi level and part of the orbitals transforms from separation to connection, manifesting the apparent redistribution of the electronic structure in SO_2_ molecule. For the interaction between the atoms in Fig. 6d, the O 2p, Zn 4s, and Zn 3d orbitals are found hybridized at multiple energy levels, such as − 6, − 4, and − 2 eV. The hybridization signifies the strong chemical interaction between O atom and Zn atom and effectively supports the formation of Zn-O bond as calculated in the optimized structure. In the SO_2_F and SO_2_F_2_ system, due to the dramatic deformation in structure, the molecular orbitals are strongly activated and redistributed with many new formed orbitals. The F 2p and O 2p orbitals in SOF_2_ are intensely hybridized with the Zn 4s and 3d orbitals at − 8, − 7, and + 3 eV. The hybridization between F atoms and Zn atoms can be identified near − 7, − 5, and + 3.5 eV. The apparent hybridization between Zn atom and the trapped F, O atoms is the evidence of the formation of stable chemical bonds, namely the Zn-F and Zn-O bonds, which can be an explanation for the strong adsorption effect between ZnO-C_3_N monolayer and the two gases. Combined with the results obtained from four adsorption systems, except for H_2_S molecule, the other three molecules (SO_2_, SO_2_F, and SO_2_F_2_) can be firmly adsorbed when exposed to the ZnO-C_3_N monolayer. This conclusion proves that the substance has the potential gas removal application in the high-voltage equipment.

### Gas Sensing Performance Evaluation

**Fig. 7:**
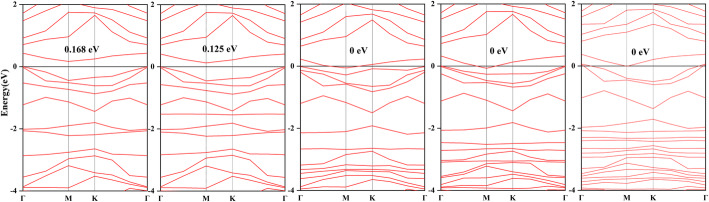
Band structure of **a** ZnO-C_3_N monolayer, **b** H_2_S system, **c** SO_2_ system, **d** SOF_2_ system, and **e** SO_2_F_2_ system

To achieve the gas detection, a moderate change in conductivity is necessary for post-adsorption resistive-type devices. The conductivity of a certain system is related to its bandgap according to the following equation [59]:

3 $$ \sigma =A\times {e}^{-{E}_g/2{k}_BT} $$

where *A* is a certain constant, *k*_B_ is the Boltzmann constant (8.62 × 10^−5^ eV K^−1^) and T is the temperature. An inversely proportional relationship can be recognized between conductivity and bandgap, the wider the bandgap, the more difficult it is for electron to cross the forbidden band. Figure 7a demonstrates that the bandgap in ZnO-C_3_N monolayer is pretty small as 0.168 eV which is less than half of the bandgap in pristine C_3_N (0.39 eV), while the properties of semiconductor and indirect bandgap remain the same that can be judged from the different location of the bottom of conduction band (M) and the top of valence band (Γ). With respect to the bandgap of adsorption systems, different variations can be found in Fig. 7b–d. In H_2_S system, the bandgap decreases to 0.125 eV on account of the downward movement in the bottom of conduction band. In other systems, the novel impurity level that appears at the top of valence band meets with the Fermi level near the M point and results in the zero bandgap of these systems, which can be considered strong p-type doping for the ZnO-C_3_N monolayer [43, 60]. Although the semiconducting nature of the adsorbed structures may be covered by their metallic-like property of zero bandgap [61], the zero bandgap could provide visible enhancement in conductivity. It is much significant to improve the response performance of the devices based on ZnO-C_3_N monolayer. To amplify further analysis of the response (*R*) performance, herein, we calculate it based on the following equation [62]:

4 $$ R=\frac{\left|\frac{1}{\sigma_{\mathrm{gas}}}-\frac{1}{\sigma_{\mathrm{gas}}}\right|}{\frac{1}{\sigma_{\mathrm{pure}}}}=\left|\frac{\sigma_{\mathrm{pure}}-{\sigma}_{\mathrm{gas}}}{\sigma_{\mathrm{gas}}}\right| $$

where *σ*_pure_ and *σ*_gas_ represent the conductivity of the ZnO-C_3_N system before and after adsorption, respectively. According to the calculations, the *R* for the H_2_S system and the rest three systems are 0.567 and 0.962, namely the conductivity would increase by 56.7% and 96.2% when the adsorption occurs on the surface of ZnO-C_3_N monolayer; in this case, it is possible to detect the existence of these gases.

The recovery time (*τ*) is another important parameter to estimate the property of sensors used in gas detection, which indicates the time spent in removing the adsorbed gas molecules. By reviewing the literature, *τ* could be calculated by the van’t Hoff Arrhenius equation [63]:

5 $$ \tau ={F}^{-1}{e}^{-{E}_{\mathrm{a}}/{k}_{\mathrm{B}}T} $$

where *F* is the attempt frequency and defined as 10^12^ s^−1^ in this study. *E*_a_ is the energy barrier for desorption which is assumed the same as the value of *E*_ads_ here, *k*_B_ and *T* are defined the same as in Eq. (3). From the Eq. (5), desorption for the adsorbed gases would be harder as the *E*_ads_ getting larger, but it can also be controlled by raising the working temperature.

Table 2 lists the recovery time required for the four gases to remove from the surface of ZnO-C_3_N monolayer. For the H_2_S molecule, the small *E*_ads_ undoubtedly reflects the low energy barrier for desorption, accordingly, causing the extra short recovery time in microseconds. At the meantime, for the systems with larger *E*_ads_, it seems impossible to separate the gas molecule from the surface at the working temperature as the desorption will takes several days. When it reaches 498 K and 598 K which can rarely occur in the electrical equipment, the desorption process could be accelerated to the minutes scale for SO_2_ and SOF_2_, respectively. The extremely strong adsorption between the gas molecules (SO_2_, SOF_2_, and SO_2_F_2_) and the surface reveals the potential application of the ZnO-C_3_N monolayer as a gas scavenger to remove the SF_6_ decomposition species and maintain the good insulation state inside the power system. In addition, in the actual structure, given the high quantity of ZnO nanocrystals on C_3_N, the effect is expected to be substantially enhanced. Besides, comparing to the original configuration, the activity of the gases releasing from the ZnO-C_3_N monolayer is greatly impaired and can hardly exert impact on the system because of the severe deformation of the molecular structures (SOF_2_ and SO_2_F_2_). In terms of the H_2_S, it is supposed that the unstable interaction and extremely short recovery time of ZnO-C_3_N monolayer towards H_2_S are unable to provide an effective detection as the adsorption density is supposed to be small.

**Table 2:** The recovery time of ZnO-C_3_N towards H_2_, CH_4_, and C_2_H_2_ at different temperature

Temperature (K)	τ-H_2_S (s)	τ-SO_2_ (s)	τ-SOF_2_ (s)	τ-SO_2_F_2_ (s)
298	3.15 × 10^−5^	4.70 × 10^8^	2.81 × 10^16^	1.33 × 10^47^
398	4.11 × 10^−7^	3.01 × 10^3^	2.00 × 10^9^	1.85 × 10^32^
498	3.07 × 10^−8^	2.34	1.06 × 10^5^	2.39 × 10^23^
598	5.45 × 10^−9^	0.02	150.29	2.90 × 10^17^
698	1.59 × 10^−9^	6.70 × 10^−4^	1.40	1.74 × 10^13^

## Conclusions

In this paper, a model of ZnO-modified C_3_N is established and the optimal structure is investigated by placing the ZnO particle on the surface of C_3_N in various orientations and position. Thus, the adsorption parameters of the ZnO-C_3_N monolayer on four SF_6_ decomposition species, namely H_2_S, SO_2_, SOF_2_, and SO_2_F_2_, were obtained by analysing the *E*_ads_, DOS, *Q*_T_, and band structure before and after adsorption. It is found that the H_2_S molecule can hardly adsorb stably on the nanostructure; at the same time, the other gases are strongly trapped in the ZnO particle. These results confirmed that the adsorption performance of ZnO-C_3_N monolayer allows its potential application as gas scavenger to sweep SO_2_, SOF_2_, and SO_2_F_2_ from the high-voltage equipment, which keeps the insulation strength and the safe operation of power system. Plus, the frontier molecular orbital theory implies that ZnO-C_3_N monolayer possesses the possibility to estimate the dielectric state of SF_6_ insulation equipment as an indicator, given the obvious changes in conductivity caused by the adsorption of the abovementioned gases.

## Supplementary information

**Additional file 1: Figure S1.** The initial positions and optimized structure of ZnO-C_3_N with the symmetry axis of ZnO vertical to the plane (O_1_). **Figure S2.** The initial positions and optimized structure of ZnO-C_3_N with the symmetry axis of ZnO vertical to the plane (O_2_). **Figure S3.** The initial positions and optimized structure of ZnO-C_3_N with the symmetry axis of ZnO parallel to the plane (O_3_).

## Data Availability

All the data and material are provided in the manuscript and supplementary file.
