# Supplementary material for: Adsorption of SF6 Decomposed Products on ZnO-Modified C3N: A Theoretical Study
Source: Nanoscale Res Lett. 2020 Sep 25;15:186. doi: 10.1186/s11671-020-03412-y (PMC7519028; doi:10.1186/s11671-020-03412-y)
Supplement: Supplementary file 1 — Additional file 1: Figure S1. The initial positions and optimized structure of ZnO-C3N with the symmetry axis of ZnO vertical to the plane (O1). Figure S2. The initial positions and optimized structure of ZnO-C3N with the symmetry axis of ZnO vertical to the plane (O2). Figure S3. The initial positions and optimized structure of ZnO-C3N with the symmetry axis of ZnO parallel to the plane (O3). [file 11671_2020_3412_MOESM1_ESM.docx]

**Supplementary file for**

**Adsorption of SF_6_ decomposed products on ZnO-modified C_3_N: A theoretical study**

PengWu^1^, Xiaoxing Zhang ^1, 2, 3*^, Dachang Chen^1^ and Ju Tang^1^.

^1^ School of Electrical Engineering and Automation, Wuhan University, Wuhan 430072, China

^2^ Hubei Key Laboratory for High-efficiency Utilization of Solar Energy and Operation Control of Energy Storage System, Hubei University of Technology, Wuhan 430068, China;

^3^ State Key Laboratory of Power Transmission Equipment & System Security and New Technology, Chongqing University, Chongqing 400044, China

***** Correspondence: Xiaoing Zhang; E-mail: [xiaoxing.zhang@outlook.com](mailto:xiaoxing.zhang@outlook.com)

**Figure S1.** The initial positions and optimized structure of ZnO-C_3_N with the symmetry axis of ZnO vertical to the plane (O_1_).

**Figure S2.** The initial positions and optimized structure of ZnO-C_3_N with the symmetry axis of ZnO vertical to the plane (O_2_).

**Figure S3.** The initial positions and optimized structure of ZnO-C_3_N with the symmetry axis of ZnO parallel to the plane (O_3_).
